# Observations on Spasmodic Constriction of the Anus

**Published:** 1819-06

**Authors:** W. White


### *Observations on Spasmodic Constriction of the Anus;* by W. White, Esq.

IN consequence of some observations which appeared in your Journal of January last, respecting a mode of treating spasmodic constriction of the anus, by Mons. Boyer, I have taken the liberty of sending you the following remarks and case, if you judge them worthy a place in your valuable Miscellany.

Some of your readers may probably recollect that a paper of mine, on Stricture of the Rectum, was noticed in the Medical and Physical Journal of April, 1818, as having been read at the anniversary meeting of the London Medical Society; and also a wish was expressed that it might be printed in the next volume of the Transactions of that Society, from the important practical remarks it contained. Reference is made to that paper, because there were likewise some observations on spasmodic constriction of the sphincter ani muscle, which will prove the complaint had not been overlooked; though, as Dr. Baillie* has very justly observed, it has been very little noticed by practitioners. But the principal design in this communication is to shew that the complaint may be relieved without the necessity of dividing the sphincter; an operation, the propriety of which I had a long time since contemplated performing in the event of other means failing that had been adopted: and I think every candid practitioner will agree with me, that, if equal benefit can be obtained by a method which does not derange the structure of the part, it ought to be preferred.

Although the spasmodic constriction alluded to is sometimes only occasional, yet in other instances the spasm becomes permanent; but even then the muscle contracts still further on attempting to introduce either the finger or a bougie into the rectum, so as always to render it extremely difficult, and on some occasions altogether impracticable, at least to introduce the finger. In those cases which have come under my notice, the sphincter has appeared much thicker and broader than it is naturally; so that the distance between the external and internal margin of the muscle (called, by Dr. Baillie, external and internal sphincter,) is much greater than in a state of health: at the same time there does not appear to be *any other alteration* in the

* A case, with some very important remarks on spasmodic constriction of the sphincter ani, is published in the fifth volume of the Transactions of the Royal College of Physicians, by Dr. Baillie.

structure of the part; for, when the finger is admissible, the inner membrane of the intestine has a healthy feel. I have also noticed the fissure mentioned by Mons. Boyer, and coincide in opinion with that gentleman. It is the consequence of the contracted state of the anus, as the effort to pass a motion in such cases is exceedingly great; and, from the violent and often ineffectual straining, the complaint in that situation is far more painful and distressing than either a spasmodic or permanent stricture higher up the passage.

In various cases, where simple stricture has occurred a few inches up the rectum, I have observed a disposition to spasmodic constriction at the anus, though in so slight a degree as not to excite the particular attention either of the patient or the surgeon; yet, however, sufficiently obvious to induce me to think, that in more violent cases of that nature, the superior stricture is commonly the cause of the spasmodic constriction which takes place at the sphincter.

In one case (at present under my care) the existence of two strictures at the rectum had been ascertained by different surgeons a long while before the patient felt any inconvenience whatever at the sphincter, which is now spasmodically constricted; and I have not hitherto met with a case of that nature which has not been accompanied by a stricture higher up the rectum: from which circumstance I have been led to believe, that, whenever there is a permanently contracted state of the sphincter, if the intestine be carefully examined with a bougie not less than ten inches in length, and of a sufficient diameter to pass the sphincter, a stricture higher up the passage will be found very often to exist. Nevertheless, the cases mentioned by M. Boyer may perhaps be considered as a proof that the spasmodic constriction is also an original disease, from the complete and permanent advantage which is said to have been derived from the division of the sphincter by the bistoury. From this difference in our experience, it would seem as if the complaint sometimes occurred as a primary, and at other times as a secondary, affection.

With respect to the mode of treating spasmodic constriction at the anus, I have to observe that the principal mean is the bougie, which has not hitherto disappointed me in removing that complaint; but it is of great consequence what kind is employed,* as those commonly sold for strictures in the rectum are too hard, and generally produce too much irritation, to be used with advantage, not only in the spasmo-

* I have given directions, in the second edition of my Work on Strictures of the Rectum, how to prepare such as I employ.

dic, but also in the simple permanent, stricture, which occurs higher up the intestine. In using the bougie, great caution is necessary, as it should be introduced in a gentle and slow manner until it passes above the upper stricture, and to remain as long as the patient can bear it, which at first, perhaps, will not be longer than a quarter of an hour or twenty minutes; but, after the passage becomes accustomed to the stimulus, the patient will be able to retain it a considerable length of time. It is, however, proper to observe, that the size of the bougie should be very gradually increased, or otherwise there may be so much inflammation excited as to render it necessary to suspend its use for some time. The plan should be regularly persevered in till the passage admits a large bougie; and I have hitherto found, that, in proportion as the intestine becomes dilated at the superior stricture, the spasmodic constriction at the anus gives way.

As the bougie, then, has been found adequate to the removal of permanent spasmodic constrictions of the sphincter ani, when combined with strictures higher up the passage, it may be reasonably expected that the same means will prove equally efficacious when the complaint occurs as a primary affection. If Mons. Boyer considers it expedient to employ a large bougie after the division of the sphincter, ("to prevent irregular adhesions of the wound,") which must be very painful, why not in the first instance give that instrument a fair trial?

CASE.

I received the following statement some time before the patient came under my care. It was written by his son, dated Nov. 7th, 1817.

"Sir,—I have collected the following particulars from my father, and those who have attended him, which I take the liberty of stating to you, and request your opinion upon them as early as you can conveniently write. The first sensible symptoms that appeared, took place about a fortnight before my father found himself obliged to confine himself at home. These were, *heat* after walking; and, after much walking, pain, which for a few days generally subsided in an hour: these symptoms, however, increased, and the voiding of his stools was followed by considerable anguish and increased heat, till, on September 3, he was incapable of going out. Piles were suspected, and the ordinary treatment adopted without success; indeed, I think his complaint was aggravated by it. His sufferings became excruciating. He had been inspected by a surgeon, who pronounced, I believe, in the first instance, the formation of matter; but subsequently, on my enquiring of him what he considered my father's complaint to be, said he feared a scirrhous tumor. He is still under the same surgeon's care, who at present enjoins nothing but injecting and guarding against costiveness. My father certainly does not suffer so much as at the first; but, upon every evacuation by stool, or passing of wind, most acute pain is produced, which sometimes lasts one or two hours, particularly when a stool has been passed. Sometimes he has two or three stools a-day, with perhaps as many mistaken calls, generally very relaxed, and always voided with sudden violence, so as, with all care, not to be able always to confine them to the utensil, but discharged against the top. Whenever they become figured, which is very rarely, they pass in the form of very narrow tape, and adhere to the back of the utensil in a plaited pile. Matter has once been seen. Gravel passes with the urine mixed with mucus. A very frequent sensation of distension in the belly, with a feeling, after every stool, of not having voided all the fæces; occasional gripings at the pit of the stomach; at passing a stool, something which my father describes as resembling a *lip** protrudes on one side of the anus. My father's age is 70-71; has been all his life subject to very relaxed bowels; for more than thirty years invariably vomited his breakfast, and often his dinner. This ceased almost entirely twenty years ago, when he was attacked, in all appearance, in a way similar to the present; but rapidly recovered, after having one day voided a stool so powerfully offensive to the smell as nearly to cause his fainting away.

"I should mention also that the passage is, *and ever has been since my father can remember*, remarkably narrow; so much so, that, whenever he passed a figured stool, and was able to see it by having used the night-table, it never seemed larger than the finger of a little child. He has led a very active, and indeed laborious, life, but regular and temperate in all his habits; and I should consider him yet as possessing much vigour of constitution for his age. I am, Sir, very truly yours."

In answer to the preceding statement, I informed the gentleman that it was impossible to give a decided opinion in such a case, notwithstanding his very accurate description of the symptoms; for, though it was evident the passage must be in a very contracted state, yet the nature of it could only be ascertained by a manual examination. The friends of the patient were anxious that he should put himself under my care; but, from the history of the case, I gave them very little encouragement to hope that I could render him any service. However, as soon as he felt himself able, he undertook a journey from London to Bath, where he arrived in the beginning of January, 1818. Although he had been relieved from the acute symptoms induced by inflammation supervening, he still continued to experience a great deal

* This was merely a small fleshy excrescence.

of pain at every evacuation; and the inclination to go to stool was so sudden and frequent, that he was afraid to go out of the house, so that the greatest part of the day was spent in the most uncomfortable and tantalizing manner, constantly feeling as if something remained which it was not in his power to discharge. He also complained of great pain and heat about the anus, and a sense of fullness in the bowels, with a considerable difficulty in passing wind downwards. His general health and appetite were good.

On examination, I found the sphincter ani contracted to such a degree as to render the introduction of the finger impracticable; however, with some difficulty and exquisite pain to the patient, a small bougie was passed into the rectum: and, between five and six inches from the anus, a stricture was discovered. On his making an effort as if going to stool, a superficial fissure was observed at the back part of the anus, which appeared to be nothing more than the skin having given way from the violent straining of the part.

The use of the bougie was proposed as the most likely means of affording relief; but I confess there was very little expectation that the patient would have submitted to its repeated introduction, from the torture it occasioned on passing through the sphincter the first time. However, the success I had met with on similar occasions, enabled me to encourage him to persevere; and, in the course of nine weeks, the passage admitted a large bougie, and the sphincter ani became so much relaxed as to allow the finger to pass very readily into the rectum, which had a healthy feel as far as the finger could reach: neither was there any enlargement of the prostate gland, which had been the opinion of another surgeon who had been previously consulted; but this opinion was given without any examination of the part, because it was at that time impracticable to introduce the finger into the rectum.

With the assistance of only a tea-spoonful of castor-oil taken daily, one or two evacuations were procured in the course of the morning, which became gradually more copious, and discharged in a more natural and regular manner; so that, in the course of a short time, having a motion was attended with very little pain or difficulty, and ultimately none at all. The tenesmus also, with which the patient had been so long annoyed, entirely ceased.

As auxiliaries, it may be right to mention that the hip-bath was used for a few minutes every day previous to employing the bougie, which appeared to render its introduction easier by somewhat relaxing the sphincter; and, after its removal, a few grains of the ext. papav. alb. dissolved in a little warm water, was thrown up as an injection, which was continued as long as the irritation of the part rendered it necessary.

As soon as the patient could properly manage the bougie himself, he returned home in a very comfortable state, to the great consolation of his friends, who had not calculated on his receiving so much benefit, particularly at his advanced period of life. He was requested to persevere in the occasional use of the bougie. I have twice heard of him since he left Bath, and the accounts both times were very satisfactory.

*Bath; April 9th,* 1810.

